# A Temporal Filter for Binaural Hearing Is Dynamically Adjusted by Sound Pressure Level

**DOI:** 10.3389/fncir.2019.00008

**Published:** 2019-02-13

**Authors:** Ida Siveke, Andrea Lingner, Julian J. Ammer, Sarah A. Gleiss, Benedikt Grothe, Felix Felmy

**Affiliations:** ^1^Department Biology II, Division of Neurobiology, Ludwig-Maximilians-Universität München, Munich, Germany; ^2^Institute of Zoology and Neurobiology, Ruhr-Universität Bochum, Bochum, Germany; ^3^Graduate School for Systemic Neurosciences, Ludwig-Maximilians-Universität München, Munich, Germany; ^4^Institute of Zoology, University of Veterinary Medicine Hannover, Hannover, Germany

**Keywords:** dorsal nucleus of the lateral lemniscus, sound pressure level, auditory filter, glutamate receptor, echo perception

## Abstract

In natural environments our auditory system is exposed to multiple and diverse signals of fluctuating amplitudes. Therefore, to detect, localize, and single out individual sounds the auditory system has to process and filter spectral and temporal information from both ears. It is known that the overall sound pressure level affects sensory signal transduction and therefore the temporal response pattern of auditory neurons. We hypothesize that the mammalian binaural system utilizes a dynamic mechanism to adjust the temporal filters in neuronal circuits to different overall sound pressure levels. Previous studies proposed an inhibitory mechanism generated by the reciprocally coupled dorsal nuclei of the lateral lemniscus (DNLL) as a temporal neuronal-network filter that suppresses rapid binaural fluctuations. Here we investigated the consequence of different sound levels on this filter during binaural processing. Our *in vivo* and *in vitro* electrophysiology in Mongolian gerbils shows that the integration of ascending excitation and contralateral inhibition defines the temporal properties of this inhibitory filter. The time course of this filter depends on the synaptic drive, which is modulated by the overall sound pressure level and N-methyl-D-aspartate receptor (NMDAR) signaling. In psychophysical experiments we tested the temporal perception of humans and show that detection and localization of two subsequent tones changes with the sound pressure level consistent with our physiological results. Together our data support the hypothesis that mammals dynamically adjust their time window for sound detection and localization within the binaural system in a sound level dependent manner.

## Introduction

The amplitude and the temporal response properties of auditory neurons depend on absolute sound pressure level (Joris and Yin, 1992; Heil and Irvine, 1997; Wallace et al., 2002; Tollin and Yin, 2005; Palmer and Shackleton, 2009). Furthermore, neuronal responses to different sound pressure levels are not static, but adapt in a context-specific manner along the auditory pathway (Dean et al., 2005; Magnusson et al., 2008; Watkins and Barbour, 2008; Wen et al., 2009, 2012; Dahmen et al., 2010; Stange et al., 2013).

To extract relevant features of an auditory signal from a constant stream of concurrent sounds and their reverberations, the auditory system is equipped with various spectral and temporal filters that dynamically process monaural or binaural signals (Olsen, 1992; Eggermont, 2011). A link between auditory filter properties and sound pressure level has been demonstrated by quantifying sound intensity dependent changes in spectro-temporal receptive fields of neurons, for instance in the inferior colliculus (Lesica and Grothe, 2008).

To understand how temporal filters within the binaural system, which is essential for sound localization and sound source segregation, depend on overall sound pressure level, we focus on the neuronal circuitry around the dorsal nucleus of the lateral lemniscus (DNLL). This auditory brainstem structure receives binaural information from the superior olivary complex (Glendenning et al., 1981; Shneiderman et al., 1999; Siveke et al., 2006; Kelly et al., 2009). In the DNLL information is encoded on the single cell level *via* large changes in firing rates (Aitkin et al., 1970; Kelly et al., 1998; Burger and Pollak, 2001; Fitzpatrick and Kuwada, 2001; Kuwada et al., 2006; Siveke et al., 2006; Pecka et al., 2010) based on α-amino-3-hydroxy-5-methyl-4-isoxazolepropionic acid receptor (AMPAR) and *N*-methyl-D-aspartate receptor (NMDAR) mediated excitation (Faingold et al., 1993; Fu et al., 1997; Kelly and Li, 1997; Wu, 1999; Kelly and Kidd, 2000; Porres et al., 2011; Ammer et al., 2012; Siveke et al., 2018). While NMDAR mediated excitation in the DNLL increases firing rates, it does not affect the timing and accuracy of the neuronal responses. In turn this NMDAR induced amplification increases the mutual information in the DNLL and enhances the neuronal code of binaural cues along the auditory pathway (Siveke et al., 2018).

The DNLL generates a reciprocal GABAergic inhibition that outlasts the sensory stimulus by several milliseconds (Yang and Pollak, 1994; Burger and Pollak, 2001; Pecka et al., 2007). On the cellular level the time course of this inhibition is based on asynchronous release, synaptic transmitter spillover and passive postsynaptic integration of hyperpolarizing inhibition (Ammer et al., 2015). This “long-lasting” (compared to the stimulus duration) inhibition suppresses lagging information and has been suggested to act as a temporal filter (Yang and Pollak, 1994; Kelly and Li, 1997; Burger and Pollak, 2001; Pecka et al., 2007; Meffin and Grothe, 2009). Specifically, the suppression of lagging information can act as a temporal low pass filter for binaural signals and thereby eliminate spurious binaural signals (Pecka et al., 2007; Meffin and Grothe, 2009). Furthermore, previous studies showed that the long-lasting inhibition in the DNLL and the corresponding suppression of neuronal activity depends on the location of the sounds (Pecka et al., 2007; Meffin and Grothe, 2009). This is coherent with the general idea of reciprocal inhibition as a neuronal circuit motif suitable for mediating context dependent adaptations (Mysore and Knudsen, 2012).

Here, we specifically asked how sound pressure level and neuronal excitability modulates the long-lasting inhibition in the DNLL, thereby testing the general hypothesis that sound pressure level causes adaptation of temporal filters in the binaural system. We show in the DNLL of adult Mongolian gerbils that the duration of this inhibitory action depends on the excitatory drive that is, in turn, based on the sound pressure level and NMDAR signaling. Furthermore, we present psychoacoustic evidence that the perception of subsequent tones in humans is dynamically adjusted in a sound level-dependent manner as predicted from our *in vitro* and *in vivo* DNLL data. Together the data support our hypothesis that the binaural system of mammals dynamically adjusts their time window for the detection and localization within the binaural system in a sound level dependent manner.

## Materials and Methods

This study was carried out in accordance with the recommendations of the German law and approved by the governing agency of Bavaria (Regirung Oberbayern) and its ethic committee with the protocol number 55.2-1-54.2531-105-10.

### *In vivo* Electrophysiology

Electrophysiological recordings were performed in 3–4 months old Mongolian gerbils (*Meriones unguiculatus*) of either sex. Animals were initially anesthetized [ketamine (20%) and xylazine (2%) diluted in 0.9% NaCl solution] with an intraperitoneal injection (0.5 ml/100 g body weight). During recording animals were continuously injected subcutaneously with anesthetics (rate of 1.7–2.4 μl/min). Body temperature was kept at 37.5°C. Ketamine/Xylazine anesthesia is supposed to reduce NMDAR signaling (Coan and Collingridge, 1987). Thus, the magnitude of the measured effects of NMDAR activation *in vivo* may represent an underestimate.

**Figure 1:**
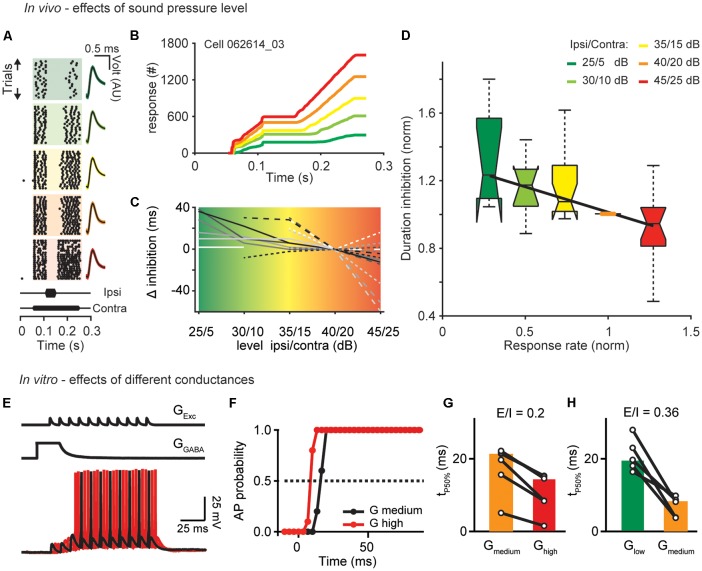
Sound level and excitatory drive set the time course of inhibition. **(A)** Single unit response raster plots to different sound levels (increasing from top to bottom). Each dot represents an action potential. On the right are shown the waveforms of the APs (black traces: mean response waveform; gray/colored envelopes: SEM; AU, arbitrary unit). **(B)** Cumulative profiles of the responses shown in (**A**; best frequency = 7,200 Hz). **(C)** Calculated change in duration relative to the control condition (40/20 dB) of all cells measured. **(D)** Boxplot of the normalized duration of inhibition as a function of normalized response rate (before the inhibitory stimulus) for different sound levels (from low to high: green, light green, yellow, orange, red). The duration of inhibition and the response rate measured under different sound level conditions was normalized to duration and response rate of the control 40/20 dB-stimulation (orange dot). The central mark is the median for each sound level (*n* = 10, 12, 19, 21, 15–from low to high level), the edges of the box are the 25th and 75th percentiles, whiskers extend to the most extreme data points. Correlations: *R* = −0.62, *p* = 0.002. **(E)** To simulate persistent inhibition (PI) *in vitro*, an artificial excitatory conductance (G_Exc_) was injected to evoke APs during the decaying phase of a GABAergic conductance (G_GABA_). Excitation was shifted relative to inhibition in 3.33 ms increments. AP probability was computed from 11 repetitions for different values of G_EXC_ (4–18 nS) at different levels of G_GABA_ (G_low_: 20 nS; G_medium_: 50 nS; G_high_: 90 nS) mimicking the different loudness conditions of the *in vivo* experiments. **(F)** Duration of AP suppression. Dotted line indicates 50% probability. Time from start of inhibitory decay to time of 50% (tP50%) AP probability were compared for different G_GABA_ at equal E/I ratios [G_GABA_ medium/orange compared to G_GABA_ high/red **(G)** and G_GABA_ low/green compared to G_GABA_ medium/orange **(H)**]. Symbols represent single cells and bars median values (*n* = 6, 7).

**Figure 2:**
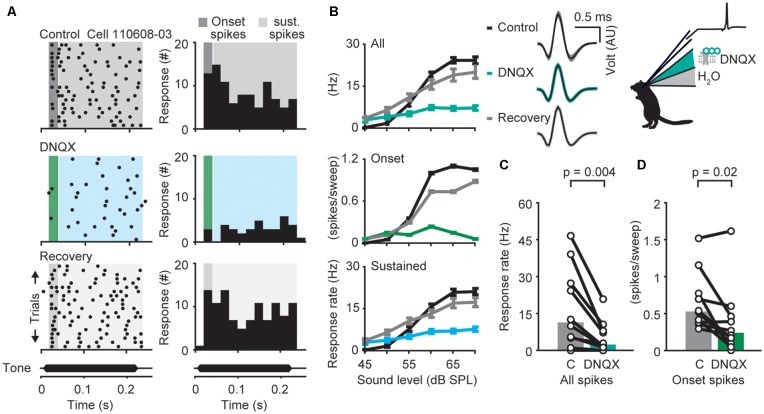
α-amino-3-hydroxy-5-methyl-4-isoxazolepropionic acid receptor (AMPAR)-dependent modulation of neuronal responses in the dorsal nuclei of the lateral lemniscus (DNLL) *in vivo*. **(A)** Response raster plots (left) and PSTHs (right) to a 200-ms tone presented at the neuron’s best frequency 25 dB above threshold (bf = 1,200 Hz). The dark gray/green area presents the time window of the onset response, the light gray/blue area presents the sustained response. **(B)** Representative average responses (10 repetitions) to different sound pressure levels of the same neuron shown in **(A)** before drug application (control, black), during presence of the AMPA antagonist 6,7-dinitroquinoxaline-2,3-dione (DNQX; upper graph: all spikes/blue-green; middle graph: on response/green; lower graph: sustained spikes/blue), and after drug application (recovery, gray). Error bars represent SEM on the right voltage responses of the recorded neuron (black traces: mean response waveform; gray/colored envelopes: SEM; AU, arbitraty unit). **(C,D)** Firing rates measured at the highest sound pressure level plotted for each cell (circles) during control condition (“C” in figure) and drug application. The population medians (colored bars) are shown before and after DNQX application for different time windows (*n* = 20). The responses are shown for the whole duration (all spikes; **C**) or only for the onset (first 25 ms) of the stimulus (onset spikes; **D**) as indicated in the raster plot in **(A)**. Significance was assessed using Wilcoxon’s signed-rank test.

**Figure 3:**
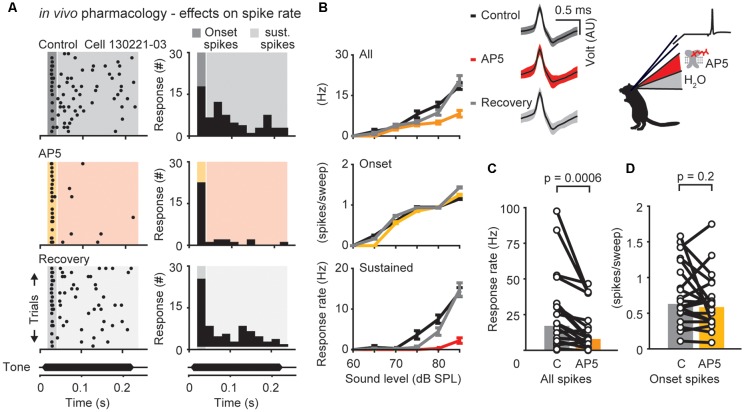
N-methyl-D-aspartate receptor (NMDAR)-dependent modulation of neuronal responses in the DNLL *in vivo*. **(A)** Response raster plots (left) and PSTHs (right) to a 200-ms tone presented at the neuron’s best frequency 25 dB above threshold (bf = 500 Hz). The dark/yellow area presents the time window of the onset response, the light gray/red area presents the sustained response. **(B)** Representative average responses (10 repetitions) to different sound pressure levels of the same neuron shown in **(A)** before drug application (control, black), during presence of the NMDAR antagonist D-(-)-2-Amino-5-phosphonopentanoic acid (D-AP5; upper graph: all spikes/orange; middle graph: on response/yellow; lower graph: sustained spikes/red), and after drug application (recovery, gray). Error bars represent SEM Upper panel on the right shows voltage responses of the recorded neuron (black traces: mean response waveform; gray/colored envelopes: SEM; AU, arbitrary unit). **(C,D)** Firing rates measured at the highest sound pressure level plotted for each cell (circles) during control condition and drug application. The population medians (colored bars) are shown before and after D-AP5 application for different time windows (*n* = 20). The responses are shown for the whole duration (all spikes; **C**) or only for the onset (first 25 ms) of the stimulus (onset spikes; **D**) as indicated in the raster plot in **(A)**.

Details of all procedures used for surgical operations, acoustic stimulus delivery, stimulus calibration, and recording techniques have been published previously (Siveke et al., 2012). Parts of the first set of neuronal data reported in Figures 1, 2 are reanalyzed data from Siveke et al. (2018). Briefly, auditory stimuli were set to the neuron’s best frequency. Monaural contralateral pure tones (200 ms tone including 5 ms cosine rise/fall times) were presented while randomly varying the sound pressure level (5 dB steps in a range of −5 to +40 dB relative to threshold). Repetition rate was 2 Hz. To quantify the time course of inhibition, a contralateral pure tone at best frequency (200 ms tone including 5 ms cosine rise/fall times) was presented 20 dB above threshold to excite DNLL neurons. This sound was paired with a short, louder ipsilateral pure tone to induce inhibition (50 ms at best frequency, 40 dB above threshold), which started 50 ms after the contralateral tone (see schema in Figure 3A). While leaving the interaural intensity difference constant, persistent inhibition (PI) was measured at five different overall sound levels (5–25 dB above threshold contralateral and 25–45 above threshold ipsilateral).

During recordings NMDARs (*n* = 31) and AMPARs (*n* = 10) were blocked using D-(-)-2-Amino-5-phosphonopentanoic acid (D-AP5; 50 mM; pH = 7.4) and 6,7-dinitroquinoxaline-2, 3-dione (DNQX, 20 mM; pH = 7.4; chemicals from BioTrend), respectively. The drugs were dissolved in dH_2_O and applied iontophoretically using multibarreled electrodes. The center barrel was filled with 1 M NaCl for current balancing. In some experiments three barrels were filled with drugs and one with a ringer solution having the same pH (7.4) as the drug solution. Retention (+20 nA) and ejection currents (−70 nA) were applied *via* a Neurophore BH-2 (Harvard/Medical Systems). The effects of the drugs on the neuronal responses were recorded during and immediately after applying the drug and during the subsequent recovery.

### *In vitro* Electrophysiology

**Figure 4:**
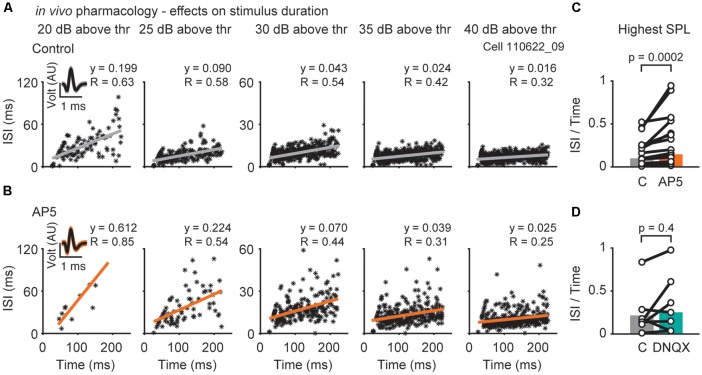
Effects of NMDAR- or AMPAR-mediated excitation on inter-spike-intervals (ISIs). **(A)** Representative responses of a single neuron (bf = 1,000 Hz) to different sound pressure levels (increasing level from left to right; 20, 25, 30, 35, 40 dB above threshold) before **(A)** and during drug (AP5) application **(B)**. Each asterisk represents the spike time and its corresponding ISI. Insets show voltage responses of the recorded neuron. **(C,D)** The resulting slope (y) of the linear regression at the highest SPL presented is plotted for each cell during control, D-AP5 **(C)** and DNQX **(D)** application (black circles). The population medians (colored bars) are shown before and during application of D-AP5 (**C**; orange, *n* = 15), or DNQX (**D**; blue-green, *n* = 7). Significance was assessed using Wilcoxon’s signed-rank test.

Female and male Mongolian gerbils of postnatal day (P) 30–36 (Figure 4) were first anesthetized and then decapitated and the brains were removed in cold dissection solution containing (in mM) 50–120 sucrose, 25 NaCl, 25 NaH_2_CO_3_, 2.5 KCl, 1.25 Na_2_HPO_4_, 3 MgCl_2_, 0.1 CaCl_2_, 25 glucose, 0.4 ascorbic acid, 3 myo-inositol and 2 Na-pyruvate, at pH 7.4 when bubbled with carbogen (95% O_2_ and 5% CO_2_). Two-hundred micrometer thick transverse slices containing the DNLL were cut with a vibratome (VT1200S Leica, Wetzlar, Germany). Slices were incubated for 45 min at 36°C in extracellular recording solution (same as dissection solution but with 125 mM NaCl, no sucrose, 1.2 or 2 mM CaCl_2_ and 1 mM MgCl_2_). All recordings were carried out at near physiological temperature (34–36°C). Cells were visualized and imaged with a microscope (BX50WI, Olympus, Hamburg, Germany) equipped with gradient contrast illumination (Luigs and Neumann, Ratingen, Germany) and a TILL Photonics system (Gräfelfing, Germany) composed of an Imago CCD-camera, a Poly-IV monochromator, and its control unit.

Recordings were performed using an EPC10/2 amplifier (HEKA Elektronik, Lambrecht, Germany). Data were acquired at 20–50 kHz and filtered at 3 kHz. In whole-cell current-clamp, the bridge balance was set to 100% after estimation of the series resistance and was monitored repeatedly during recordings. The liquid junction potential was calculated and corrected for according to Barry (1994), with a custom-written IGOR Pro script on the basis of ion concentrations (Ammer et al., 2015). The internal solution consisted of (in mM): 145 K-gluconate, 4.5 KCl, 15 HEPES, 2 Mg-ATP, 2 K-ATP, 0.3 Na_2_-GTP, 7.5 Na_2_-Phospocreatine, 5 K-EGTA (pH 7.3, liquid junction potential 17 mV). One-hundred micrometer Alexa 488 or 568 were added to the internal solution to control for cell type and location.

Simulated conductances were injected with an analog conductance amplifier (SM-1, Cambridge Conductance, Royston, UK) with the reversal potential of inhibition and excitation set to the measured −90 and estimated 0 mV, respectively (Ammer et al., 2015). Conductance waveforms were constructed artificially in IGOR Pro (WaveMetrics, Lake Oswego, OR, USA). Excitatory conductances were constructed according to the AMPAR kinetics in P23–26 animals with decay time constants of τ = 2 ms (Ammer et al., 2012). EPSG trains consisted of 11 individual EPSGs waveforms of equal amplitude which were varied from 4 to 18 nS. Inhibitory conductance was applied with amplitudes of 20, 50 or 90 nS decaying from a constant plateau with a biexponential function possessing a weighted time constant of τ_w_ = 10.1 ms, determined following train stimulation in the DNLL of P30–36 animals (Ammer et al., 2015). Action potential probability was sampled every 3.3 ms with 10 repetitions at three time-shifts and inter-trial intervals of 2 s. All waveforms were scaled and saved as HEKA templates using a customary modified PPT script (Mendez and Würriehausen, Göttingen). To suppress synaptic inputs, conductance clamp recordings were performed in the presence of 0.5 μM Strychnine, 10 μM SR95531, 20 μM DNQX, and 10 μM *R*-3-(2-Carboxypiperazin-4-yl)propyl-1-phosphonic acid (CPP).

### Psychophysics

The psychophysical experiment was similar to that published by Pecka et al. (2007) except that the loudspeaker location was modified to match the present experimental setup (see Figure 6A). Nine human listeners (five males and four females, mean age 23.7 ± 3.9 years) participated voluntarily. All subjects showed normal hearing at audiometric frequencies between 250 and 8,000 Hz. Listeners were seated in a double-walled, sound-attenuated, anechoic chamber (Industrial Acoustics Company GmbH, Niederkrüchten, Germany), lined on all surfaces with 20 cm acoustic foam wedges. Sounds were presented in the free sound field *via* two loudspeakers (CANTON Plus XS.2, CANTON Elektronik GmbH and Co. KG, Weilrod, Germany) mounted at ±40° to the left and the right of the listener’s midline. The distance between each loudspeaker and the listener’s head was approximately 1 m. Every loudspeaker was equalized with an individual finite-impulse-response filter resulting in a flat frequency and linear phase response from 100 Hz to 20 kHz as measured with a 1/2–inch microphone (B&K 4189) positioned in the middle of the subjects’ interaural axis.

**Figure 5:**
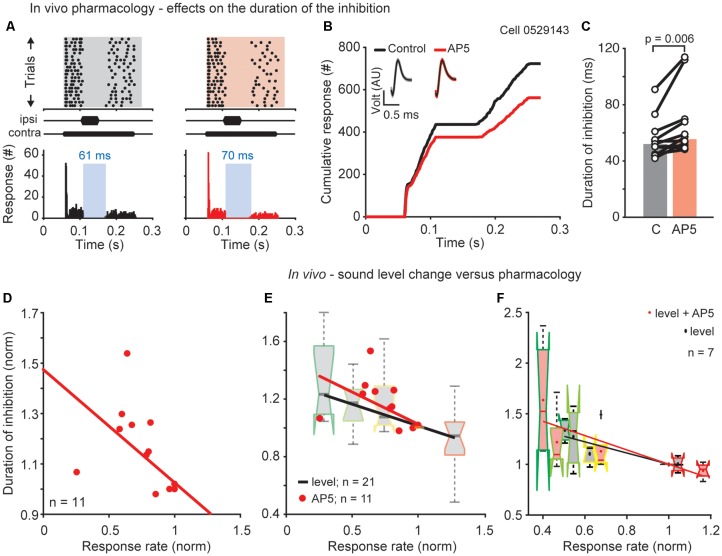
NMDAR mediated effects on the duration of the DNLL specific PI. **(A)** Raster plots (top) and corresponding PSTHs (bottom) before (left) and during D-AP5 application (right/red) and their cumulative profiles (**B**; best frequency = 2,600 Hz). Insets: mean waveform (black) and SEM (gray/red envelopes). AU, arbitrary unit. **(C)** Duration of inhibition for each cell (circles) and the population medians (bars; *n* = 11) for control and D-AP5 conditions. Significance was assessed using Wilcoxon’s signed rank test. **(D)** Normalized duration of inhibition as a function of normalized response rate (before the inhibitory stimulus). Each dot represents the normalized duration and response rate of a single neuron. For each neuron the duration of inhibition and response rate measured after blocking the NMDAR current was normalized to the duration of the inhibition and the response rate under control conditions. Correlation: *R* = −0.996, *p* = 0.0003. **(E)** Comparison of the drug and sound level experiments showing the normalized duration of inhibition as a function of normalized response rate for the two different experiments plotted together. Data are replotted from **(D)** and Figure 1C. **(F)** Results of a subpopulation (*n* = 7) where both experiments, drug and different sound levels, were performed in the same neuron (correlations level: *R* = −0.91, *p* = 0.03; correlations AP5: *R* = −0.85, *p* = 0.066).

**Figure 6:**
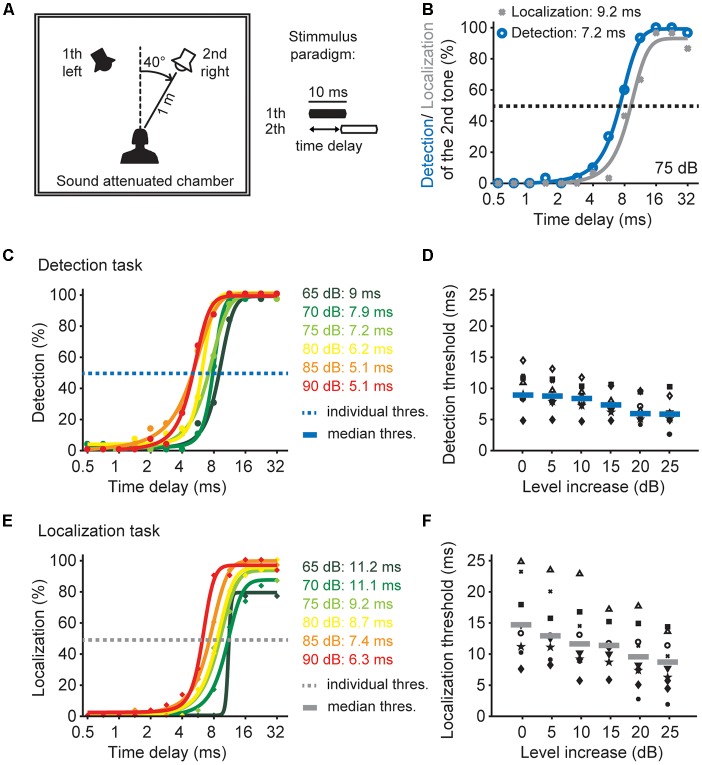
Sound pressure level effects on time window of the detection and localization of two tones. **(A)** Schematic drawing of the experimental design and the paradigm. **(B)** Psychophysical performance of one listener in the detection task (blue) and the localization task (gray) at 75 dB SPL. **(C)** Psychophysical performance of one listener in the detection task of different sound pressure levels. Dots depict the performance as a percentage, solid lines represents sigmoid fits. The time delay of the detection threshold of the 2nd tone was determined at 50% performance (blue dashed line). **(D)** Detection thresholds as a function of presented sound level for all listeners (*n* = 10). Different black symbols represent individual listeners; blue lines represent median detection thresholds. **(E)** Psychophysical performance of one listener in the localization task of different sound pressure levels. Dots depict the performance in percent, solid lines represents sigmoidal fits. The time delay of the localization threshold of the 2nd tone was determined at 50% performance (gray dashed line). **(F)** Localization thresholds as a function of presented sound level for all listeners (*n* = 9). Different black symbols represent individual listeners; gray lines represent median localization thresholds.

All stimuli were generated in MATLAB at a sampling rate of 44.1 kHz, and were digital-to-analog (D/A) converted (MOTU 24I/O, MOTU Inc., Cambridge, MA, USA) and amplified (NAD CI9120, NAD Electronics International, ON, Canada) before being presented to the listeners. The stimulus paradigm consisted of a leading sound always presented from the left side and a lagging sound presented from the right side of the listener’s midline (please see Figure 6A). Both sounds were bursts with a length of 10 ms (2 ms rise and fall time) and a frequency of 4 kHz. The time delay between the left and right sounds was randomly chosen from 13 different increments between 0.5 and 32 ms. The stimulus level for each sound pair presentation was randomly chosen from six different levels between 65 and 90 dB SPL in 5 dB steps.

Listeners were asked to perform two different tasks: the detection task and the localization task. For the detection task listeners indicated whether they heard one or two sounds. For the localization task listeners indicated whether the second sound was perceived at an indicated location. Each listener completed 30 trials for each time delay and each sound level. Performance was then averaged over these 30 trials and a sigmoid function was fitted to the data. This was done individually for each listener and sound level. The detection threshold and the localization threshold were defined as the time delay at 50% performance. Median thresholds for each sound level were then calculated across all listeners.

## Results

### Sound Pressure Level Modulates the Time Scale of Inhibition in the DNLL

To test the hypothesis that temporal filters adjust to sound pressure level, we investigated the influence of sound intensity on the duration of inhibition in the DNLL. To this end, we performed extracellular *in vivo* recordings and applied a 200 ms tone stimulus to the contralateral ear to excite DNLL neurons while presenting an additional shorter 50 ms tone to the ipsilateral ear to evoke inhibition [see schematic drawing in Figure 1 of Pecka et al. (2007)]. To test the effects of the absolute sound pressure level, the sound pressure level was changed while keeping the difference in sound level between the long excitatory (contralateral) and the short inhibitory (ipsilateral) tones (i.e., the interaural level difference, ILD) constant. We found that the duration of inhibition is longer than the stimulus duration and considerably increases further when the absolute sound pressure level was decreased (Figures 1A,B). Interestingly, the onset of the inhibition does not change with the sound pressure level. The change of duration of inhibition with decreasing sound pressure level was analyzed in a population of DNLL neurons (*n* = 21, Figure 1C). Even if not seen for all sound pressure levels all neurons showed a tendency to increasing duration of the inhibition with decreasing sound level. To quantify this effect over the population of cells recorded, we normalized the sustained response rates before the ipsilateral, inhibitory stimulation under different sound pressure level conditions to the response rate under control condition (condition 40/20 dB) and the duration of the inhibition to the duration of inhibition under control condition (Figures 1A,B orange line, Figures 1C,D orange dot). The normalized firing rate changes induced by different sound pressure levels significantly correlated with the normalized duration of inhibition [Figures 1C,D; level: *R* = −0.62, *p* = 0.002, *n* = 21, correlation coefficient *t*-test (matlab)].

To attain a cellular-level understanding of the sound-level-induced changes of the inhibition observed *in vivo*, we performed conductance clamp recordings *in vitro*. Our *in vivo* data show that the time of onset of inhibition stayed constant but the duration of inhibition changed with the sound pressure level at the end of the ipsilateral stimulation. Therefore, we focused in our *in vitro* experiments on the termination of the inhibitory action. Previous studies showed that the prolonged inhibition in the DNLL is mediated by long lasting GABAergic inhibition (Pecka et al., 2007; Ammer et al., 2015). Following the previous studies inhibitory GABA conductances were simulated with a weighted decay time constant of τ = 10 ms and paired with the injection of an excitatory conductance train of 11 consecutive EPSC-like waveforms simulating the activity of ipsilateral afferent fibers (Figure 1E, Ammer et al., 2015). To mimic the different sound pressure levels in the *in vivo* recordings, different conductance levels were now applied. The conductance amplitudes of excitation and inhibition were co-varied while keeping the excitation-inhibition ratios unchanged (modeling the effect of constant ILDs in the *in vivo* experiments). This allowed us to quantify the duration of action potential suppression, hence the inhibitory action (Figure 1F). Higher overall conductance values at equal E/I ratios resulted in shorter durations of action potential suppression (Figures 1G,H). Thus, larger conductance levels reduce the duration of inhibition.

### Time Course of AMPA Receptor Mediated Excitation in the DNLL

Next, we investigated the contribution of the two major excitatory inputs to the temporal filtering in the DNLL. Neuronal excitation in adult DNLL neurons is synaptically mediated by AMPAR and NMDARs (Fu et al., 1997; Wu, 1999; Kelly and Kidd, 2000; Porres et al., 2011; Ammer et al., 2012; Siveke et al., 2018). While fast AMPAR mediated currents are thought to mediate rapid and temporally restricted voltage responses, slow NMDAR mediated currents can support long lasting depolarizations. Since the time course of synaptically driven excitation is critical for the temporal characteristics of the neuronal filters, we compared the effects of different glutamate receptor mediated excitation on the sound evoked onset and sustained responses *in vivo* (Figures 2, 3). Single unit recordings were performed before, during and after iontophoretic application of DNQX, an AMPAR antagonist (Figure 2), or D-AP5, a specific NMDAR antagonist (Figure 3). The rates of action potential firing in response to pure tones presented at different sound levels (rate level functions) were assessed during the presence and absence of antagonists. Typical single cell responses before (upper panels), during, or directly after application of DNQX (middle panels), and after recovery (lower panels), are shown in Figure 2A. DNQX application significantly and reversibly decreased mean firing rates in response to a stimulus 25 dB above threshold over the whole response window by about 73% (Figure 2B, upper panel) from 11 to 2 Hz (Figure 2C; *p* = 0.004, *n* = 10, Wilcoxon’s signed rank test). Since the contribution of AMPAR mediated currents to spike generation may change during the course of tone stimulus, we segregated the drug effects for the onset (green, Figure 2B, middle panel) and the sustained (blue, Figure 2B, lower panel) component of the response (see Figure 2A, shadowed background). As exemplified in Figure 2B, the AMPAR antagonist DNQX significantly decreased the onset and the sustained component of the mean response rate (Figure 2D; control = 26 Hz, DNQX = 12 Hz, *p* = 0.02, *n* = 10), Wilcoxon’s signed rank test. In other words, AMPARs are crucial for driving supra-threshold responses in DNLL neurons throughout sound stimulation.

### Time Course of NMDA Receptor Mediated Excitation in the DNLL

The presence of NMDAR mediated excitation in adult DNLL neurons has been clearly demonstrated (Fu et al., 1997; Wu, 1999; Kelly and Kidd, 2000; Porres et al., 2011; Ammer et al., 2012; Siveke et al., 2018), however its specific impact on the response rate at different time windows during sound stimulation has not been determined. Thus, action potential firing was assessed before (control = black; Figures 3A,B) and after iontophoretically applying D-AP5 (AP5 = red; Figures 3A,B). Measured over the whole response window (onset spikes and sustained spikes) D-AP5 significantly reduced the mean firing rate at 25 dB above threshold by about 53% from a median of 17–8 Hz. (Figure 3C, *p* = 0.0006, *n* = 20, Wilcoxon’s signed-rank test). In contrast to the AMPAR antagonist application, the onset component was not significantly reduced after D-AP5 application (Figures 3B,D; control = 31 Hz, D-AP5 = 29 Hz, *p* = 0.2, *n* = 20, Wilcoxon’s signed-rank test). Thus, NMDARs are activated to support action potential generation during ongoing sound stimulation.

The temporally restricted impact of NMDAR activation becomes even more apparent when comparing spike-frequency adaptation (Figures 4A,B). Blocking NMDARs specifically reduced the number of action potentials in the later part of the response, which leads to larger inter-spike-intervals (ISIs) towards the end of the stimulus (Figure 4C; slope of the regression line: control = 0.10, D-AP5 = 0.15, *p* = 0.0002, *n* = 15, Wilcoxon’s signed rank test). Block of AMPARs had no effect on spike-frequency adaptation (Figure 3D; control = 0.21, DNQX = 0.25, *p* = 0.4, *n* = 7, Wilcoxon’s signed-rank test). Taken together, AMPARs and NMDARs act together to shape sound evoked output in DNLL neurons. AMPARs are effective throughout the sound stimulation while NMDARs contribute predominantly to the ongoing, sustained component of the sensory evoked response. Thus, NMDAR mediated excitation is suitable to affect the slow temporal filtering in the DNLL.

### NMDAR Mediated Excitation in the DNLL Affects the Duration of Inhibition

So far we have shown that the prolonged inhibition in the DNLL negatively correlates with the sustained response rate and that this sustained response rate depends on NMDARs. To correlate these findings we now assess the role of NMDARs on temporal filtering and measured their influence on overcoming the inhibition in the DNLL. We performed extracellular recordings *in vivo* and applied the same stimulus as used before (Figures 1A–C), while blocking NMDAR mediated excitation at the site of recording. The decreased excitation induced by D-AP5 application led to a prolongation of the inhibition visible in the raster plot (Figure 5A, top), PSTH (Figure 5A, bottom) and cumulative response plot (Figure 5B). The longer plateau of the cumulative plot indicates the longer inhibitory action. A significantly prolonged duration of inhibition after blocking NMDARs was found in 9 of 11 cells (Figure 5C, from 52 to 56 ms, *p* = 0.006, *n* = 11). Since we found, that the neurons that showed the greatest reduction in response rate after blocking NMDARs also tended to show the greatest increase in the duration of inhibition, we examined whether the firing rate in general correlates with the duration of inhibition. Therefore, we normalized the response rate and the duration of inhibition after blocking NMDAR currents to the response rate and duration of inhibition under control conditions before blocking. The firing rate and the duration of inhibition correlated significantly (Figure 5D; *R* = −0.996, *p* = 0.0003, *n* = 11, correlation coefficient *t*-test). This finding indicates that the duration of inhibitory action is altered post-synaptically by the strength of excitation driven through NMDARs.

Next, we compared the relationship between the duration of inhibition and the firing rate in the two sets of *in vivo* experiments (Figures 1C, 5D). From the NMDAR-experiment shown in Figure 5 and the sound-pressure-level-experiment shown in Figure 1, the same relationship between the decreases in duration of inhibition with the increasing in response rate was obtained (Figure 5E). This similarity was directly confirmed in a subset of neurons (Figure 5F insert; *n* = 7) for which both experimental paradigms were combined and the inhibition at different absolute sound pressure levels with and without blocking NMDARs was recorded (level: *R* = −0.91, *p* = 0.03; drug and level: *R* = −0.85, *p* = 0.065, correlation coefficient *t*-test). These results suggest that the duration of inhibitory action strongly depends on the excitatory synaptic drive.

### The Psychophysical Time Window for Detecting Sounds Depends on Overall Sound Level

We now know that the inhibitory temporal filter generated in the binaural DNLL circuitry is adjusted to the overall sound pressure level. Furthermore, there is evidence that this binaural circuitry could be the means for the elimination of spurious and/or lagging information from echoes or multiple active sound sources (Yang and Pollak, 1994; Kelly and Li, 1997; Burger and Pollak, 2001; Pecka et al., 2007; Meffin and Grothe, 2009). Since our *in vivo* data predict a change in the temporal filter by sound pressure level, we hypothesized that such dynamic adjustments can be detected in a psychophysical experiment. To understand the psychophysical consequences we used an established experimental task to assess the time window for detecting and localizing multiple inputs (Blauert, 1997) at different intensities (Figure 6A). Human listeners were presented with two sounds in a free field environment and asked two different questions: first, can you hear two tones (detection task) and second if you hear a second sound, do you localize the second tone at a distinct position from the first (localization task). These two-tone tasks are ideal to test the temporal filters of the binaural auditory system because, when two sounds are presented, the delay between the sounds is important for the individual detection and localization percept they induce (Blauert, 1997). If the delay is short, we only hear the first sound and the percept of the second sound is suppressed. If the delay is increased, we perceive a second sound, but not its distinct location. The complete percept, detection and localization of the second sound, is only reached at longer delays. These different time delays for the detection and localization are apparent for an example subject (Figure 6B). The listeners detected the presence of both sounds at a delay of 7.2 ms (Figure 6B, blue line) but were only able to localize the second sound at a distinct position with a 50% correct responses at a time delays larger than 9.2 ms (Figure 6B, gray line).

In previous studies Shinn-Cunningham et al. (1993) and Goverts et al. (2000) proposed a relation between such “dominance of preceding signals” and sound pressure level. Therefore, we determined the time window for detection and localization of two sounds using a range of different sound pressure levels. The detection performance at different absolute sound levels for one human listener and the detection thresholds (at 50% performance) for all listeners are shown in Figures 6C,D. With increasing sound level the median detection threshold decreased from 9 ms at the lowest sound pressure level to approximately 5 ms at the loudest sound pressure level. This reduction demonstrates that the minimal time delay to detect two separate sounds decreases with increasing sound pressure level.

The localization performance at different absolute sound levels for one human listener and the localization thresholds (at 50% performance) for all listeners are shown in Figures 6E,F. With increasing sound level the median localization threshold decreased from 15 ms to approximately 9 ms at the highest sound pressure level. Thus, the ability to localize a second sound at a distinct location is increased for louder sounds. This reduction demonstrates that the second sound is perceived at the right location at smaller time delays between the two sounds when the sounds are louder. Comparing the two tasks shows that the time delay threshold to detect two sounds is lower than the threshold to localize a second sound (Figure 6D vs. Figure 6F). Furthermore the time window for the detection and localization threshold systematically decreases with sound level intensity. Taken together, these psychophysical data from human listeners show that both detection and localization thresholds of two subsequent sounds strongly depend on the absolute sound pressure level.

## Discussion

Here, we present evidence that binaural filtering in the DNLL is adjusted in a dynamic manner. First, AMPAR and NMDAR mediated excitation in the DNLL is temporally distinct. While AMPAR mediated excitation is mainly present at the beginning of the stimulus, NMDAR mediated excitation supports sustained supra-threshold activity during the ongoing part of the stimulus. Second, the duration of long-lasting inhibition depends on the overall excitation of the postsynaptic neuron and is modulated by sound pressure levels. From our findings we hypothesize that the inhibition in the DNLL acts as a dynamic temporal filter that suppresses information in a sound level dependent manner *via* NMDAR mediated excitation. Third, we corroborate this hypothesis with human psychophysics revealing a similar time window for sound level dependent detection and localization of multiple sounds.

Using *in vivo* pharmacology we show that fast AMPAR mediated excitation acts throughout the time course of sound stimulation, while NMDAR mediated excitation predominantly effects action potential generation during ongoing sounds. In this way, NMDARs reduce firing rate depression during ongoing sound presentation, thereby promoting high firing rates. Since NMDAR mediated excitation is a key element for adjusting the high firing rate, we reasoned that it might be crucial for filtering binaural responses in the DNLL. Indeed, the contribution of NMDARs to postsynaptic excitation limits the duration of contralaterally provided inhibitory action. Thus, NMDAR activation is a key element for the filter properties of DNLL neurons, and we speculate that it adjusts the time window for the processing of lagging signals. Thus, we extend the knowledge about the presence of NMDA receptors in the DNLL and their impact on binaural processing in the DNLL (Faingold et al., 1993; Fu et al., 1997; Kelly and Li, 1997; Wu, 1999; Kelly and Kidd, 2000; Porres et al., 2011; Ammer et al., 2012; Siveke et al., 2018) by demonstrating that NMDARs additionally play a role in temporal filtering.

The overall sound level enhances both the excitatory drive and the duration of the inhibition in the DNLL. Thus, loud sounds will drive strong inhibition but also strong excitation. Despite the sound level dependent increase in inhibition the increase in excitation dominates and reduces the duration of inhibitory action. This scenario is consistent with our conductance clamp recordings that demonstrate that with the same balance of excitation and inhibition, the overall conductance level results in different duration of inhibition. Low sound pressure levels lead to a long duration of inhibitory action and high sound pressure levels to a short duration of inhibitory action. Taken together, the duration of inhibition, which forms the basis of the temporal filter in the DNLL is dynamically adjusted by the activation of NMDARs. Moreover, this indicates that the postsynaptic firing rate accurately reflects the ongoing subthreshold excitatory drive, which depends on acoustic context.

The decrease of inhibitory duration observed in the gerbil *in vivo* physiology was compared to the decrease in detection and localization threshold with increasing sound level in human listeners. There are several limitations comparing this electrophysiological data measured in the gerbil with the human psychophysical experiments. The most obvious difference is the use of different species. However, gerbils are an ideal animal model to study complex spatial hearing especially in regard to two tone suppression, as even the localization dominance of the first sound is comparable to humans (Wolf et al., 2010) and the detector for low frequency sound sources appears equally sensitive to humans (Lingner et al., 2012). Another limitation is that our experimental designs are not directly comparable. We measured electrophysiologically in the DNLL the duration of inhibition following a (first) tone. In the psychophysical task the duration of inhibition following the first tone was only indirectly measured by investigating the time window of the ability to detect and to localize the second tone. Nevertheless, the decrease in detection and localization threshold with increasing sound level in human listeners show the same trend as the decrease of the duration of inhibition observed in the gerbil *in vivo* physiology. This finding is coherent with the idea that the duration of the inhibition in the DNLL plays an important role in adjusting auditory filters for the perception of multiple sounds. Although the neuronal basis for the suppression of multiple sounds and echoes is still debated and different aspects may be processed at different levels of the auditory system (Clifton, 1987; Brown et al., 2015), our data is consistent with an important role for the DNLL (Pollak et al., 2003; Pecka et al., 2007). In line with previous data that suggested a relationship between the dominance of preceding signals and sound pressure level (Shinn-Cunningham et al., 1993; Goverts et al., 2000), we here describe that the suppression of a lead-lag perception in human listeners is adjusted depending on sound level. Therefore, we propose that the long-lasting inhibition in the DNLL acts as a temporal filter that suppresses the binaural perception of multiple sounds or echoes in a sound level dependent manner.

In our psychophysical experiments, we focused on a two-tone paradigm investigating the ability to detect and localize a second sound lagging a preceding sound. This probability we found to be increased with increasing pressure level. The results do not exclude the possibility that in general the detection and spatial discrimination of sound sources may increase with sound pressure level. Here basic processing, besides the dynamics of DNLL filtering, such as increased entrainment or higher spike probability may also increase the discrimination ability. Furthermore, recent studies showed that some dynamic adjustments take place and interfere with the sound localization ability of the second tone (Lingner et al., 2018). Such basic and or adaptive mechanisms were not in the focus of this study.

We observed both shorter temporal echo detection thresholds in humans and shorter durations of action potential suppression *in vivo* and *in vitro* for increased levels of synaptic drive. Functionally we speculate that this level dependance might result in a distance filter, since the overall sound pressure level of the first stimulus and the lagging echo could contain information about the distance of the sound source. Louder stimuli generally represent closer sound sources whereas far away sound sources are generally quieter. The sound pressure dependance of echo suppression might account for these distance dependent differences, which could set the ethological objective for such distance filtering.

## Data Availability

The data that support the findings to this study are available from the corresponding authors upon request.

## Author Contributions

IS, JA and FF: conceptualization and writing—original draft. Design, acquisition, analysis and interpretation of the specific experiments: IS and SG (*in vivo* recordings), JA and FF (*in vitro* recordings), AL (human psychophysics). IS, JA, FF, AL and BG: writing—review and editing.

## Conflict of Interest Statement

The authors declare that the research was conducted in the absence of any commercial or financial relationships that could be construed as a potential conflict of interest.

## Abbreviations

AMPAR: alpha-amino-3-hydroxy-5-methyl-4-isoxazolepropionic acid receptor
C: control
CPP: 3-(2-Carboxypiperazin-4-yl)propyl-1-phosphonic acid
DNLL: dorsal nucleus of the lateral lemniscus
D-AP5: D-(-)-2-Amino-5-phosphonopentanoic acid
DNQX: 6,7-dinitroquinoxaline-2,3-dione
NMDARs: N-methyl-D-aspartate receptors
PI: persistent inhibition.
